# Teacher Autonomy Support in Physical Education Classes as a Predictor of Motivation and Concentration in Mexican Students

**DOI:** 10.3389/fpsyg.2019.02834

**Published:** 2019-12-20

**Authors:** Erasmo Maldonado, Jorge Zamarripa, Francisco Ruiz-Juan, Rosana Pacheco, Maritza Delgado

**Affiliations:** ^1^Facultad de Organización Deportiva, Universidad Autónoma de Nuevo León, San Nicolás de los Garza, Mexico; ^2^Facultad de Ciencias del Deporte, Universidad de Murcia, Murcia, Spain; ^3^Facultad de Psicología, Universidad Autónoma de Nuevo León, San Nicolás de los Garza, Mexico

**Keywords:** self-determination theory, autonomy support, motivation, concentration, physical education, Mexico, invariance, gender

## Abstract

There is a strong belief that physical education can affect an individual’s physical activity, healthy habits, and behaviors through pleasant, positive, and significant exercise experiences, a practical knowledge base, and comprehensive teaching strategies. However, a crucial cognitive aspect for the effective and significant learning of the activities offered in the educational environment is the concentration of students. This study aims to test a hypothetical model based on self-determination theory to assess the degree of support prediction provided by the teacher for student autonomy in the various types of motivation and on student concentration in physical education classes in high schools within the Mexican context and test invariance across gender groups. This study included 859 students between 11 and 16 years from different high schools in the city of San Nicolás de los Garza, Nuevo León (México). The Learning Climate Questionnaire, the Perceived Locus of Causality, and the Concentration scale adapted to physical education and translated into Mexican Spanish were used. Results showed good internal consistency for all instruments. Both the measurement model and the structural equation modeling showed satisfactory adjustment indexes. The results revealed that the autonomy support positively predicted autonomous motivation, controlled motivation to a lesser extent, and amotivation negatively. Furthermore, the students’ concentration was highly and positively predicted by autonomous motivation, by controlled motivation to a lesser extent, and by amotivation negatively. The model predicted 39% of variance of autonomous motivation with large effect size (ƒ^2^ = 0.64), 2% of controlled motivation with small effect size (ƒ^2^ = 0.02), 8% of amotivation with small effect size (ƒ^2^ = 0.09), and 49% of concentration with large effect size (ƒ^2^ = 0.96). Finally, the invariance analysis revealed that the model fit was invariant across gender groups. The results of this study emphasize how important it is for teachers to adopt an interpersonal style of autonomy support to generate a motivational climate that influences the concentration of students. This could contribute to the achievement of the purposes and educational objectives of the physical education class, which, in turn, might be conducive to students adopting healthy lifestyles in adolescence and beyond.

## Introduction

Physical education (PE) is regarded as one of the most viable vehicles to promote an active and healthy lifestyle, as it is able to reach a large number of children, adolescents, and youth (Pate et al., 1995; Sallis and Owen, 1999; McKenzie, 2001).

There is a strong belief that PE can affect an individual’s leisure time physical activity through pleasant, positive, and significant exercise experiences, a practical knowledge base, and comprehensive teaching strategies (Vilhjalmsson and Thorlindsson, 1998; Fox and Harris, 2003). Moreover, in theory, it has been established that individuals who went through the stage of adolescence adopting healthy habits and behaviors in a successful manner are more likely to have a longer and healthier life (Williams et al., 2000). However, to promote engagement in physical activity among diverse individuals participating in PE classes successfully, the content offered therein must be learned in a significant manner.

Concentration is a crucial cognitive aspect for the effective and significant learning of the activities offered in the educational environment. According to the American Psychological Association (2009), concentration is the act of bringing together or focusing, as, for example, bringing one’s thought processes to bear on a central problem or subject. It refers to the ability of drawing attention to a single object, and this skill may be difficult to acquire since the mind tends to shift focus every time a new stimulus is presented (Murray, 2002).

For some years, teachers and researchers have sought to maximize the time during which students focus on class activities for the purposes of optimizing learning (Berliner, 1990). Unfortunately, students do not always turn their attention to the contents of a class, as their interests, skills, and efforts differ from each other. Thus, it is interesting to understand the role of PE teachers in motivational processes and the impact they have on student concentration.

A theoretical approach that has contributed to understanding how these motivational processes occur in different contexts is the self-determination theory (SDT; Deci and Ryan, 1985, 2002; Ryan and Deci, 2017). This theory explains that motivation is multidimensional as individuals are generally motivated by a combination of diverse factors. It postulates that a person’s behavior may be intrinsically or extrinsically motivated or amotivated. These three types of motivation vary in their level of self-determination (i.e., sense of freedom to do whatever you want to do). Moreover, these types can be placed on a continuum of self-determination where the behavior would fluctuate from high levels of autonomy (i.e., intrinsic motivation), going through the mid-levels (i.e., extrinsic motivation), and on to the lowest levels (i.e., amotivation; Deci and Ryan, 1985, 2002).

Intrinsic motivation is the most self-determined type of motivation. It involves behaving in a certain manner simply for the pleasure and satisfaction of doing so. This type of motivation is an important construct that reflects the natural human interest in learning and assimilating (Ryan and Deci, 2000a). It is characterized by a high level of autonomy, and it represents the prototype of self-determined behaviors (Ryan and Deci, 2000b).

Extrinsic motivation refers to performing an activity because of the incentives or consequences associated with it. The least self-determined regulation is the external one and involves the specific manner in which someone behaves to receive a reward or avoid punishment. Introjected regulation takes place when an activity is carried out to avoid blame or to boost the ego. Identified regulation is a bit more self-determined than the introjected regulation, as it is produced when the behavior is regarded as important for the subject’s purposes. Finally, integrated regulation is the most self-determined type of extrinsic motivation, occurring when the result of the behavior is consistent with the individual’s values and needs.

The use of these regulations has also been proposed, grouping them in a broader sense to form *autonomous motivation*, derived from combining intrinsic, integrated, and identified regulations, and *controlled motivation*, resulting from the combination of introjected and external regulations (Deci and Ryan, 2000).

The last self-determined dimension is amotivation, which is present when individuals fail to perceive the contingencies between actions and their results, that is, they do not perceive the basis of their reasons. Therefore, they doubt their actions, creating a feeling of incompetence, which will likely make them give up in the future (Pelletier et al., 1999).

Due to the aforementioned points, motivation is a well-known concept for all individuals taking on leading roles such as teachers, which involves mobilizing others to act (Deci and Ryan, 1985). In PE classes, the teacher’s role is a key element that must be considered, given that students’ willingness and motivation to gain knowledge, and the possibility of acting on this basis, may result in an involvement that leads to the successful pursuit of healthy lifestyles (Johnson et al., 2011).

Students need help shifting from their dependence on the teacher to their independence in class, developing the understanding, competence, and trust required to be active in an autonomous way. This is something that must be taught, rather than waiting for it to happen *per se* (Harris, 2000).

Within SDT, autonomy support from teachers represents acts or instructions to identify, encourage, and develop internal motivational resources such as their interests, preferences, goals, and psychological needs (Assor et al., 2002; Reeve, 2006). According to Deci and Ryan (1985), individuals will improve their learning quality if they are intrinsically motivated to learn. Similarly, the conditions or contexts that provide information and autonomy support foster student learning.

In the context of PE, different studies have examined the predictive role of autonomy support on the various types of motivation through diverse cultures. According to the results obtained in these studies, autonomy support predicts the most autonomous regulations (i.e., intrinsic and identified) in a positive manner. On the other hand, autonomy support predicts the least autonomous ones (i.e., introjected external regulations and amotivation) in a negative manner (Standage et al., 2005, 2006, 2012; Standage and Gillison, 2007; Zamarripa et al., 2016; Behzadnia et al., 2018; Fin et al., 2019).

However, studies that have examined the background on student concentration during PE classes are still limited. In England, Standage et al. (2005) conducted a study to examine the model of motivation as a mediator between autonomy and concentration, among other resultants based on the SDT; the participants included 950 high school students (boys = 443; girls = 490; *M*_age_ = 12.14). They used the Learning Climate Questionnaire (LCQ) by Williams and Deci (1996), which is composed of 15 items suited to the PE class to measure autonomy support. The Perceived Locus of Causality Scale (PLOC), designed by Goudas et al. (1994), was used to assess the motivational regulations, and six items were designed to evaluate the level at which students remain focused during class activities. The instrument’s reliability analysis to measure autonomy support yielded a result of 0.96. As regards the sub-scales to measure the various motivation types, the alpha values ranged from 0.69 to 0.88. For the concentration scale, the alpha value was 0.84. The predictive character of autonomy support can be observed in its results, positively in meeting requirements (i.e., autonomy, relationships, and competence) and in intrinsic motivation. On the other hand, it showed negative results regarding external regulation and amotivation. Finally, a significant and positive connection between intrinsic motivation and concentration can be observed, as well as a significant and negative link between amotivation and concentration.

For their part, Zhang et al. (2012) examined the correlations among autonomy support, competence, beliefs associated with expectations, homework’s subjective values, concentration, and persistence/effort in 273 high school students from the southeastern United States. Autonomy support was measured using six items from the Health Care Climate Questionnaire by Williams and Deci (1996), which obtained an alpha value of 0.91, whereas the concentration level during the class was measured using the six items developed in the study conducted by Standage et al. (2005), achieving an alpha value of 0.80. The results showed correlations between the PE teacher’s autonomy support and concentration (*r* = 0.43, *p* < 0.05). Moreover, a hierarchical regression analysis was conducted, including expectancy and value constructs in the first stage, which accounted for 32.5% of concentration variance. Subsequently, when the constructs of teacher support—competence support and autonomy support—were included in the second stage, the variance percentage increased by 5.0%. These findings provide evidence with respect to the teacher’s role in building motivational constructs (i.e., beliefs related to expectation and subjective values of homework) and predicting a student’s concentration and effort.

In line with Ntoumanis and Standage (2009), physical ability, interest levels, and efforts invested by students in PE classes may be quite different depending on the subjects and cultures. Therefore, it is interesting to understand how the motivational processes are produced and the impact they have on student concentration.

**Figure 1:**
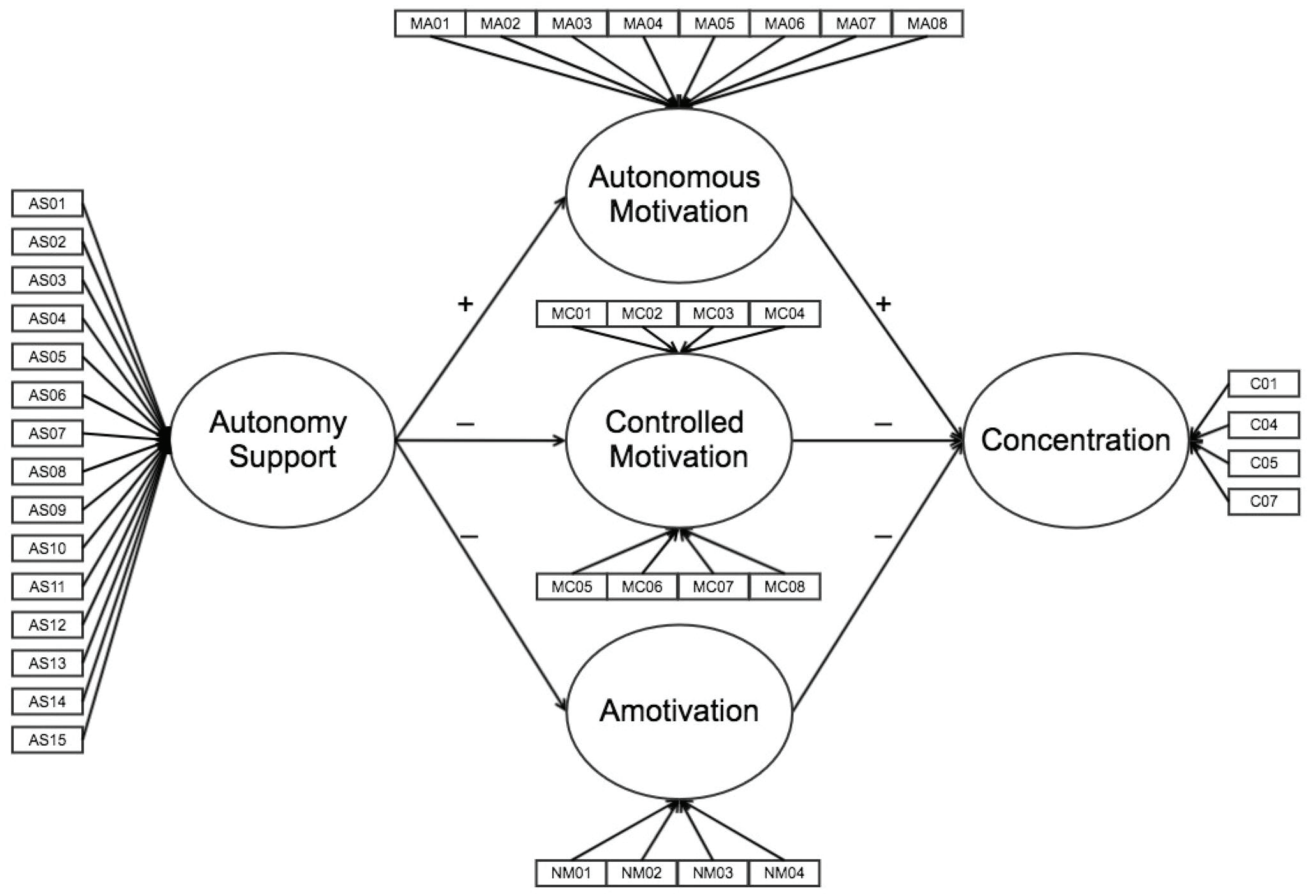
Structural model hypothesized of the relationships between autonomy support, types of motivation, and concentration.

This study aims to test a hypothetical model (see Figure 1) based on SDT (Deci and Ryan, 1985, 2002; Ryan and Deci, 2017) to assess the degree of support prediction provided by the teacher for student autonomy in the various types of motivation and on student concentration in PE classes in high schools within the Mexican context and test invariance across gender groups.

## Materials and Methods

### Design and Type of Study

The present study is an empirical research of associative strategy with explicative aim (purpose), cross-sectional with latent variables (Ato et al., 2013).

### Participants

This study involved students from 84 high schools that are part of the municipality of San Nicolás de los Garza, Nuevo León (México). The data were obtained through the Department of Public Education for the state of Nuevo León, corresponding to the 2012–2013 school year. The sample size was determined to obtain a sampling error of ±3% and a 95% confidence interval. This was a probabilistic, multi-phased cluster, and stratified sampling procedure with proportional allocation, considered by the various strata of grade, type of center, age, school shift, and sex.

The sample composed of 859 students (51% boys and 49% girls; *M*_age_ = 13.69 years old; SD = 0.98; range = 11–16) from different public (81.6%) and private (18.4%) high schools in the city of San Nicolás de los Garza, Nuevo León (México). In total, 35% of the students were in first grade, while 31.1% were in second grade and 33.9% in third grade. Most of them attended school during the morning shift (56.6%) compared to the evening (43.3%).

### Instruments

A version of the LCQ, originally designed by Williams and Deci (1996), adapted to PE by Standage et al. (2005), translated into Mexican Spanish, and validated by Maldonado et al. (2017), was used. The questionnaire included 15 items that are grouped in a single factor to measure student perception on the autonomy support offered by the teacher. The instrument begins with the following title: “In this physical education class ….” An example of an item is: “…we feel that the teacher gives us choices and opportunities.” Students use a Likert scale to answer, ranging from 1 (completely disagree) to 7 (completely agree). The results of the reliability of the autonomy support scale adapted to PE (Standage et al., 2005) showed a suitable internal consistency (*α* = 0.85).

The PLOC (Goudas et al., 1994) was used to measure the different types of regulations, adapted to PE by Standage et al. (2005), and validated for the Mexican context by Zamarripa et al. (2016). The scale comprises 20 items, four for each of the five sub-scales: intrinsic motivation, identified regulation, introjected regulation, external regulation, and amotivation. Students answered the scale items preceded by the phrase: “I take part in this physical education class….” Some examples of this scale include: “because it is funny” (intrinsic motivation), “because it is important for me to do it correctly” (identified regulation), “because I will feel bad about myself if I do not do it” (introjected regulation), “because I will get into trouble if I do not do it” (external regulation), and “but I am really not sure why I do it” (amotivation). Students used a 7-point Likert scale to answer, ranging from 1 (completely disagree) to 7 (completely agree). To create the autonomous motivation variable, the items corresponding to intrinsic motivation and the identified regulation scale were combined. On the other hand, to form the controlled motivation, the items corresponding to introjected and external regulation scales were combined. The results of the reliability of the intrinsic motivation (*α* = 0.88), identified regulation (*α* = 0.86), introjected regulation (*α* = 0.69), external regulation (*α* = 0.81), and amotivation (*α* = 0.84) adapted to PE (Standage et al., 2005) showed a suitable internal consistency. Likewise, the reliability of the autonomous motivation (*α* = 0.88), controlled motivation (*α* = 0.86), and amotivation (*α* = 0.84) in the Mexican context (Zamarripa et al., 2016) showed a suitable internal consistency.

The concentration scale developed for PE by Standage et al. (2005) and validated for the Mexican context by Maldonado et al. (2014) was used to measure students’ concentration levels. The questionnaire includes four items assessing the level at which students perceive their concentration during the PE class. These four items are grouped in a single factor to measure the students’ concentration level. The instrument begins with the following title: “For the following items, please indicate the frequency with which you feel like this during your PE class.” An example of an item is as follows: “I pay attention during the class.” Students use a Likert scale to answer, ranging from 1 (Never) to 5 (Always). The results of the reliability of the concentration scale adapted to PE (Standage et al., 2005) showed a suitable internal consistency (*α* = 0.84).

### Procedure

Authorization was requested through official letters sent to school district authorities and to each educational center principal, explaining the research objectives and the procedure that would be carried out together with the instrument’s model. This was followed by the request for authorization for implementation of the group teachers for the selected students, considering the main inclusion criteria: being regular students attending the corresponding grade, attending regular PE classes (at least twice a week), knowing or identifying their PE teacher, and being willing to complete the questionnaire. Students were informed of the study’s aim, its voluntary nature, the absolute confidentiality of the responses, and the handling of data. Moreover, they were told that there were no correct or incorrect answers and that it was mandatory to provide truthful and honest answers. The questionnaire was answered in an anonymous fashion, self-administered, and implemented collectively in the classroom during school hours. To unify the data collection conditions, the survey-takers received preparatory training. All subjects gave their parents and guardians’ written and informed consent in accordance with the Helsinki Declaration. This research was conducted in compliance with international ethical standards, which are consistent with the recommendations outlined by the APA. Ethical approval for the study was obtained from the Universidad Autónoma de Nuevo León (Mexico) ethics review committee (No. 16CI19039021).

### Statistical Analysis

First, confirmatory factor analyses (CFA) were carried out in each instrument separately. A single-factor model (autonomy support) was tested for the Learning Climate Questionnaire adapted for physical education (LCQ-PE), whereas the PLOC tested a three-factor model (i.e., autonomous motivation, controlled motivation, and amotivation) and a single-factor model (i.e., concentration) was tested for concentration. Considering the number of response categories of the observable variables (*k* ≥ 5) and the value range of skewness and kurtosis (from −1.77 to 0.95 and from −2.13 to 2.58, respectively), the maximum likelihood (ML) method was implemented, using a polychoric correlation matrix and asymptotic covariances as input (Finney and DiStefano, 2006). The fit of models was assessed through different adjustment indexes: the non-normed fit index (NNFI), which allows for the adjustment of the model’s parsimony; the comparative fit index (CFI), which estimates the relative population decrease obtained; and the root mean square error of approximation (RMSEA). CFI and NNFI values above 0.90 indicate an acceptable adjustment (Hu and Bentler, 1995). Values ranging from 0.05 to 0.10 are considered acceptable for RMSEA, and values of 0.08 or lower are considered satisfactory (Cole and Maxwell, 1985).

Second, descriptive, normality, and reliability analyses of the scales were conducted using Cronbach’s *α*, Composite Reliability (CR), and the Average Variance Extracted (AVE). Third, the hypothesized structural equation modeling (Figure 1) was tested, following the suggested step approximation by Anderson and Gerbing (1988). The first step consists of examining a measurement model to determine whether the indicators (i.e., the latent variable items) correlate with their factors in a satisfactory manner. It is vital for the measurement model to show satisfactory adjustment indexes to conduct the hypothesized structural equation model successfully. In the second step, the structural model is assessed, analyzing the general adjustment using the goodness-of-fit indexes. The maximum likelihood (ML) method was implemented, using a polychoric correlation matrix and asymptotic covariances as input, using the LISREL 8.80 program (Jöreskog and Sörbom, 2006). The effect size was calculated following the guide developed by Selya et al. (2012). Finally, the invariance of the proposed model across gender groups was tested using multi-sample invariance analysis. In order to test differences between models, a modeling rationale was considered. Differences not larger than 0.01 between NNFI and CFI values (ΔNNFI and ΔCFI) and differences not larger than 0.015 between RMSEA values (ΔRMSEA) are considered as an indication of negligible practical differences (Widaman, 1985; Cheung and Rensvold, 2002; Chen, 2007).

## Results

### Confirmatory Factorial Analysis

The CFA results showed satisfactory goodness-of-fit indexes for the single-factor model of the LCQ-PE (SB*χ*^2^ = 363.83; gl = 90; *p* < 0.001; NNFI = 0.985; CFI = 0.987; and RMSEA = 0.060), for the PLOC-PE three-factor model (SB*χ*^2^ = 982.01; gl = 165; *p* < 0.001; NNFI = 0.964; CFI = 0.969; and RMSEA = 0.076), and for the concentration questionnaire’s single-factor model (SB*χ*^2^ = 13.23; gl = 2; *p* < 0.01; NNFI = 0.980; CFI = 0.993; and RMSEA = 0.080).

### Descriptive Statistics, Normality Tests, Reliability Analyses, Convergent, and Discriminant Validity

The results of K-S with Lilliefors (1967) correction normality tests were significant for all variables excepted controlling motivation, which indicated non-normal distribution of data. However, the skewness and kurtosis values for all variables of study (see Table 1) and the items that compose them (the skewness values ranged from −1.77 to 0.95, and the kurtosis values ranged from −2.13 to 2.58) showed a moderately non-normal distribution (skew <2, kurtosis <7; Finney and DiStefano, 2006).

**Table 1:** Descriptive statistics, alpha, test of normality, and correlations between autonomy support, motivational types, and concentration.

Study variables	*M*	SD	*S*	*K*	K-S *Z*	Range	*α*	1	2	3	4
1. Autonomy support	5.19	1.22	−0.92	0.30	3.37^**^	1–7	0.92	1			
2. Autonomous motivation	5.80	1.24	−1.57	2.26	5.06^**^	1–7	0.93	0.55^**^	1		
3. Controlled motivation	4.04	1.49	−0.12	−0.84	1.26	1–7	0.84	0.15^**^	0.23^**^	1	
4. Amotivation	2.68	1.79	0.87	−0.49	5.12^**^	1–7	0.87	−0.17^**^	−0.28^**^	0.38^**^	1
5. Concentration	3.99	0.76	−0.67	0.22	3.21^**^	1–5	0.79	0.42^**^	0.58^**^	0.17^**^	−0.22^**^

*M = mean; SD = standard deviation; *S* = skewness; *K* = kurtosis; K-S *Z* = Kolmogorov-Smirnov *Z* score with Lilliefors correction*.

* *p < 0.05*;

** *p < 0.01*.

The reliability analyses showed good internal consistency for all instruments used in this study, which ranged from 0.92 to 0.79. The composite reliabilities for all instruments ranged from 0.84 to 0.94, which are considered satisfactory. In general, the AVEs for all instruments of this study were above the recommended threshold of 0.50, ranged from 0.57 to 0.73, except for the value of 0.49 for autonomous support and 0.44 for the controlled motivation dimension. These results support adequate convergent validity of the instruments.

Table 1 shows that students perceive high autonomy support from their teacher and that the activities in PE classes are mostly carried out with autonomous motivation, followed by moderate controlled motivation and low amotivation. Moreover, students show a high level of concentration during class.

### Structural Equation Modeling

First, the measurement model was tested, considering autonomy support, autonomous motivation, controlled motivation, amotivation, and concentration in PE classes as latent variables. All of them have their items as indicators, previously mentioned in the section “Instruments.” The model showed satisfactory goodness-of-fit indexes (SBχ^2^ = 2376.42; gl = 692; *p* < 0.001; NNFI = 0.98; CFI = 0.98; and RMSEA = 0.05), thus confirming the diverging validity of the latent variables.

**Figure 2:**
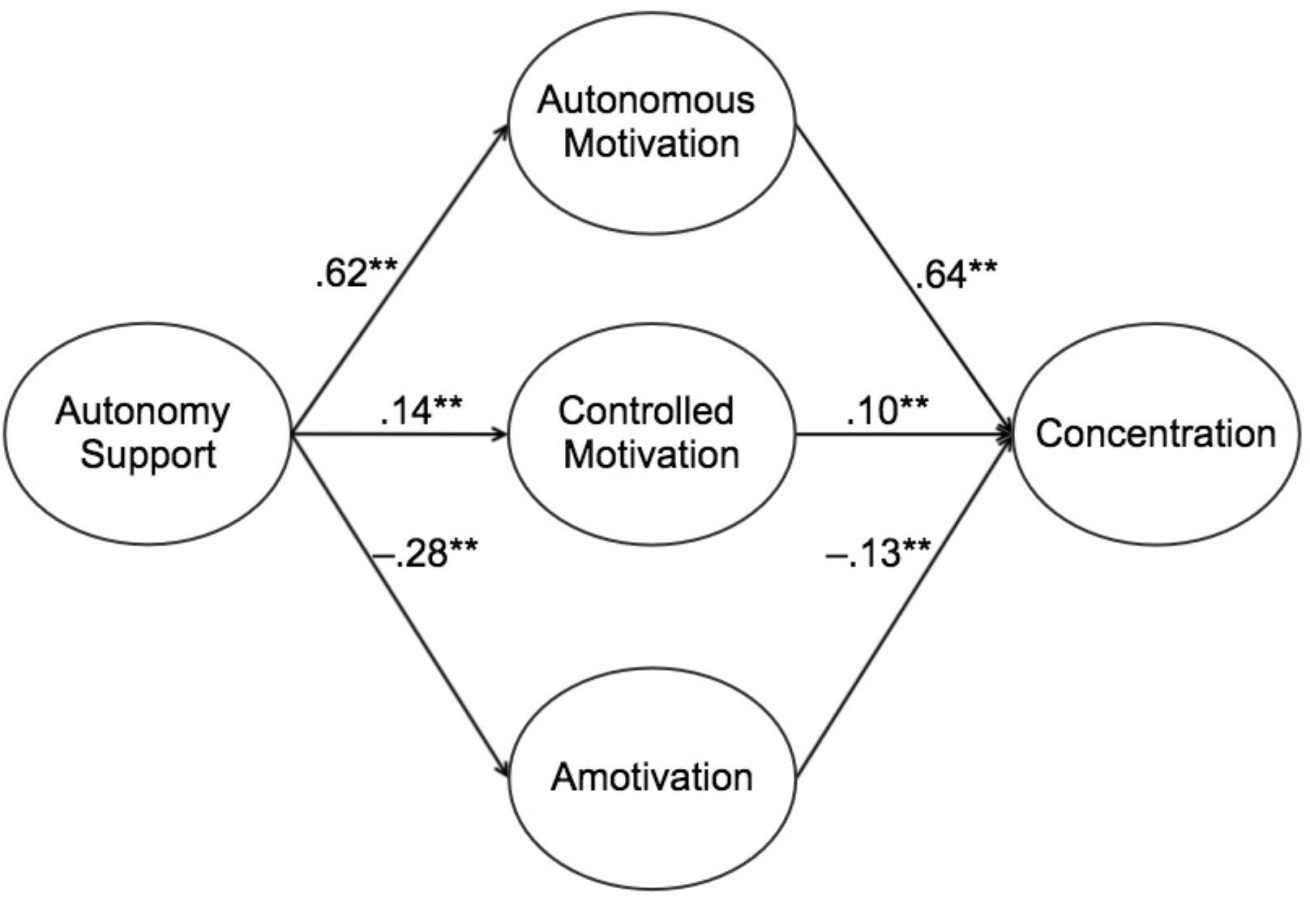
Standardized solution of the relationships between autonomy support, types of motivation, and concentration. **p < 0.01.

Later, the structural equation modeling proposed was tested (Figure 1). The goodness-of-fit indexes were satisfactory (SBχ^2^ = 2575.45; gl = 696; *p* < 0.01; NNFI = 0.97; CFI = 0.98; and RMSEA = 0.06). The autonomy support’s interpersonal style predicted positively the autonomous motivation, and this, in turn, predicted the concentration in PE classes in a positive manner. For its part, autonomy support also predicted the student’s controlled motivation, although to a lesser degree, which was also the case between controlled motivation and student concentration. Finally, autonomy support negatively predicted the amotivation, which was also predicted the concentration in the class in a negative manner (see Figure 2).

Besides the direct effects mentioned in Figure 2, the model also showed the indirect effects of autonomy support on concentration (*β* = 0.45, *p* < 0.01) through different types of motivation. As a whole, the model predicted 39% of variance of autonomous motivation with large effect size (ƒ^2^ = 0.64), 2% of variance of controlled motivation with small effect size (ƒ^2^ = 0.02), 8% of variance of amotivation with small effect size (ƒ^2^ = 0.09), and 49% of variance of concentration with large effect size (ƒ^2^ = 0.96) according to Cohen’s (1988) guidelines.

### Multi-sample Invariance Analysis

First, the model (Figure 2) was tested independently for boys (M0a) and girls (M0b) students (see Table 2). Then, a baseline multi-sample structural equation model testing for the structural invariance of the relationships among gender groups (M1) was run. Finally, a multi-sample total invariance model (M2) was run. In M2, the parameters of the structural model (i.e., those parameters that specify the relationships between the latent variables of the model) were constrained to be equal across gender groups.

**Table 2:** Results of the SEM multi-sample invariance analysis across gender.

Model	Model description	df	*χ*^2^	RMSEA	90% CI	NNFI	CFI	ΔNNFI	ΔCFI	ΔRMSEA
M0a	Boys model	1	4.65^*^	0.092	0.022–0.182	0.885	0.989			
M0b	Girls model	1	2.56	0.061	0.000–0.158	0.972	0.997			
M1	Structural invariance (baseline model)	6	27.57^**^	0.092	0.059–0.128	0.917	0.975			
M2	Total invariance model	15	72.36^**^	0.095	0.073–0.117	0.911	0.933	0.006	0.042	0.003

* *p < 0.05*;

** *p < 0.01*.

As can be seen in Table 2, the fit indices for the boy (M0a) and girl (M0b) groups were closer to or better than the values suggested by Hu and Bentler (1995).

Regarding the model that tested the structural invariance (M1), the goodness-of-fit indices were satisfactory: *χ*^2^(6) = 27.57, *p* < 0.01; NNFI = 0.917; CFI = 0.975; and RMSEA = 0.092 (90% CI = 0.059–0.128). This indicates that the pattern of relationships among the variables in the study appeared to be invariant across male and female students.

Finally, the model that tests the total invariance (M2) also showed satisfactory goodness-of-fit indices *χ*^2^(15) = 72.36, *p* < 0.01; NNFI = 0.911; CFI = 0.933; and RMSEA = 0.095 (90% CI = 0.073–0.117). When comparing the baseline model (M1) with the total invariance model (M2), the incremental fit indices indicated negligible practical differences based on ΔNNFI and ΔRMSEA values. Additionally, the modification fit indices did not indicate any structural parameter that should be set free in order to improve the fit of M2 model. Then, we considered reasonable to conclude that the parameters of the structural model were equal across gender groups. Thus, the invariance of the proposed model across gender groups was supported.

## Discussion

This study aimed to test a hypothetical model (Figure 1), based on the SDT (Deci and Ryan, 1985, 2002; Ryan and Deci, 2017), to examine the level of prediction of the support provided by teachers to student autonomy on the various motivation types, and the latter on student concentration in PE classes in high schools in the Mexican context and test invariance across gender groups.

The results of Kolmogorov-Smirnov tests indicate non-normal distribution of data, and this could be a limitation of our study; however, some authors have argued that with large sample sizes, the tests will be significant even if there are only mild deviations from normality (Ntoumanis, 2001, p. 52). Therefore, the analysis on the distribution of the data was complemented with the values of skewness and kurtosis. Unfortunately, there is no clear consensus regarding an “acceptable” degree of non-normality. Studies examining the impact of univariate normality on ML-based results suggest that problems may occur when the values of skewness are grater to 2 and univariate kurtosis are grater to 7, respectively (e.g., Chou and Bentler, 1995; Curran et al., 1996). In the present study, the skewness and kurtosis values for all observables variables showed a moderately non-normal distribution (skew <2, kurtosis <7; Finney and DiStefano, 2006); moreover, the number of response categories for all the variables was ≥5. For this reason, the maximum likelihood (ML) method was implemented, since it has been proven that if the observed data have many categories (e.g., at least five ordered categories) and are approximately normal, use of ML estimation techniques does not result in severe levels of bias in fit indices, parameter estimates, or standard errors. Problems begin to emerge as the number of response options decreases or the observed item distributions diverge widely from a normal distribution (Finney and DiStefano, 2006).

The results of the structural equation model showed that when teachers develop activities that facilitate autonomy support, that is, when they allow students to ask for things in their interest during class and even give their opinions and make decisions, where appropriate, it results in a positive relation with autonomous motivation. This, at the same time, allows the autonomous motivation to cause positive effects such as concentration during class. This result is consistent with the results obtained for other populations, such as British (Standage et al., 2005) and American (Zhang et al., 2012). A positive relationship between autonomy support and autonomous motivation was observed in both studies. At the same time, they were found to be positively associated with student concentration. Similarly, the predictive effect of autonomy support as social context in PE classes was also reviewed by Standage et al. (2006). This study focused on the satisfaction of needs and self-determined behaviors, including an assessment by teachers regarding the behavior of each student in their class. The results also showed positive relationships, consistent with the findings in the Mexican context, where this model has been developed.

In our study, contrary to what had been expected in the hypothetical model, autonomy support turned out to have a positive association with controlled motivation that, in turn, is positively associated with student concentration. Although the strength of the association was extremely low, it achieved significant levels. These results are consistent with those presented by Black and Deci (2000) and Standage et al. (2005), who state that the highest level of student concentration in class activities occurs when teachers design activities for students to perceive autonomy. They also state the support provided by teachers to student autonomy who may not reduce external pressures experienced by students to conduct activities in the academic field (e.g., guilt, obligation, and punishment).

In our study, autonomy support was negatively associated with amotivation, which was negatively associated with concentration. This suggests that when teachers provide autonomy support, that is, when students ask about things of their interest during class, give their opinions, and make decisions, the lack of intent to participate and the incompetence perceived decrease. In other words, amotivation is avoided, which in turn increases student concentration. Similar results have been found in the literature (Standage et al., 2005), even at low levels of amotivation, in line with this study.

In general, the goodness-of-fit indexes obtained from the structural equation modeling show a satisfactory adjustment of data to the hypothetical model. The results support the hypothesis that claims that the autonomy support style predicts autonomous motivation and amotivation in a positive and negative manner, respectively. The latter is widely supported and documented in various studies (Standage et al., 2005, 2006, 2012; Standage and Gillison, 2007; Zamarripa et al., 2016; Behzadnia et al., 2018; Fin et al., 2019). For their part, autonomous motivation and amotivation predicted concentration in a positive and negative manner, respectively. Although this variable has not been widely studied in the PE context, our results are consistent with the limited literature that exists (Standage et al., 2005).

Finally, the invariance analysis revealed that the model fit was invariant across gender groups. These results are consistent with those presented by Standage et al. (2005) and support the self-determination theory hypothesis that claims that the need for autonomy be equally important for males and females across cultural (Deci and Ryan, 1985, 2002; Ryan and Deci, 2017).

## Conclusions

In conclusion, presently, the content of the PE subject for Mexican basic education is being extensively discussed. The results of this study highlight the importance of knowing and adopting the autonomy support style through teacher training and education, which would facilitate the creation of learning environments that promote autonomous motivation and concentration to learn PE content, as well as avoiding amotivation, in other words, the loss of interest and motivation in class.

The main limitations of this study focus mainly on the sample’s specific characteristics as it involves high school students and is a cross-sectional study. Nevertheless, these limitations suggest possible directions for future research, as it would be interesting to extend the study with elementary school students and conduct a longitudinal collection of data to strengthen conclusions on the prediction relationships of variables included in this study.

Furthermore, the practical implications of this work focus on the design of training and education programs that guide physical educators to plan, structure, and develop classes using an autonomy support style for students to participate in class activities through the most autonomous regulations. This will ensure that students achieve positive experiences, leading to their genuine interest in and focus on learning the content of PE classes, and adopt healthy lifestyles outside the school.

## Data Availability Statement

The datasets generated for this study are available on request to the corresponding author.

## Ethics Statement

Ethical approval for the study was obtained from the Universidad Autónoma de Nuevo León (Mexico) ethics review committee (No. 16CI19039021). Written informed consent was obtained from the parents of all participants.

## Author Contributions

JZ, EM, and FR-J conceived the hypothesis of this study. EM and RP participated in data collection. JZ and MD analyzed the data. All authors contributed to data interpretation of statistical analysis. JZ and EM wrote the paper with significant input from JZ. All authors read and approved the final manuscript.

### Conflict of Interest

The authors declare that the research was conducted in the absence of any commercial or financial relationships that could be construed as a potential conflict of interest.
